# MR elastography datasets including phantom, liver, and brain

**DOI:** 10.1038/s41597-025-05968-9

**Published:** 2025-10-23

**Authors:** Yuan Feng, Suhao Qiu, Runke Wang, Shengyuan Ma, Fuhua Yan, Guang-Zhong Yang

**Affiliations:** 1 School of Biomedical Engineering, Shanghai Jiao Tong University, Shanghai, 200030 China; 2 Institute of Medical Robotics, Shanghai Jiao Tong University, Shanghai, 200040 China; 3 National Engineering Research Center of Advanced Magnetic Resonance Technologies for Diagnosis and Therapy (NERC-AMRT), Shanghai Jiao Tong University, Shanghai, 200040 China; 4 Department of Radiology, Ruijin Hospital affiliated to Shanghai Jiao Tong University School of Medicine, Shanghai, 200025 China

**Keywords:** Computational biophysics, Biomedical engineering

## Abstract

The *in vivo* characterization of biomechanical properties in soft biological tissues offers critical insights for both scientific research and clinical diagnostics. Magnetic resonance elastography (MRE) is a noninvasive technique that enables 3D measurements of the biomechanical properties of various soft tissues. While numerous inversion algorithms have been developed based on wave fields from MRE, robust and multi-parameter estimation of biomechanical properties remains an area of active development. Here we present comprehensive MRE datasets, including phantom, human liver, and human brain data. The phantom data serves as a benchmark for validation, while the liver and brain datasets represent typical application scenarios for MRE. All wave images were acquired using 3 T scanners, ensuring high-quality data. Additionally, a state-of-the-art inversion algorithm, the Traveling Wave Expansion-Based Neural Network (TWENN), is also provided for comparative analysis. These datasets provide a diverse range of application scenarios, facilitating the development and refinement of MRE inversion algorithms. By making these resources available, we aim to advance the field of MRE research and improve the inversion of biomechanical parameters.

## Background & Summary

Magnetic resonance elastography (MRE) is a noninvasive way to measure biomechanical properties of soft biological tissues^1–3^. It works by inducing shear waves within the tissues at a specific frequency, and the resulting displacement is measured using accumulated phases with motion-encoding gradients. Biomechanical properties, such as viscoelasticity, are then derived by analyzing the wave field with specialized inversion algorithms^4^. Initially developed for the clinical diagnosis of liver fibrosis^5^, MRE has since been applied to a wide range of organs and diseases. These include other abdomen organs such as pancreas^6,7^ and spleen^8–10^, neurodegenerative diseases^11^ particularly Alzheimer’s disease^12,13^ and Parkinson’s disease^14,15^, oncological applications spanning brain tumors^16,17^ and hepatic tumors^18^.

Among the many applications and studies of MRE, various inversion algorithms have been employed. The most commonly used algorithms are Direct Inversion (DI), based on the Helmholtz equation^19,20^ and local frequency estimation (LFE)^21^. Building on DI and LFE, several advanced algorithms have been proposed, such as Multifrequency Elasticity Reconstruction using Structured Sparsity and ADMM (MERSA)^22^, MRE Inversion by Compressive Recovery (MICRo)^23^, and Enhanced Complex Local Frequency (EC-LFE)^24^. To address the noise sensitivity of DI-based methods, techniques like Multifrequency Dual Elasto-Visco inversion (MDEV)^25^, k-MDEV^26^, and Elastography Software Pipeline (ESP)^27^ were developed to improve the recovery of anatomical details. Additionally, Non-Linear Inversion (NLI) algorithms based on Finite Element (FE) method^28–30^ significantly enhance estimation accuracy by iteratively updating material properties^31^. Recently, Machine Learning (ML) and Deep Learning (DL) based methods have been developed using either real-value^32,33^ or complex-valued neural network^34^. For compression wave filtering, the curl-based decomposition method remains the gold-standard approach^30,35^. This well-established technique effectively isolates shear waves by eliminating compressional wave components through its inherent divergence-free property. However, recent advances in deep learning have introduced a paradigm-shifting alternative: rather than filtering compression waves prior to inversion, state-of-the-art neural networks can be trained to inherently account for these patterns during the inversion process itself^36^. This machine learning approach incorporates comprehensive compression wave physics directly into the training datasets, enabling the algorithm to learn their characteristic signatures and appropriately compensate during reconstruction. As the research on inversion algorithm continues to expand, particularly in anisotropic and heterogeneous estimation^31,37^, benchmarking and comparison with state-of-the-art inversion datasets have become increasingly important.

Current MRE datasets for the development and comparison of inversion algorithms typically include simulated data, phantom data, and soft tissue data. As an initial step in algorithm development and validation, simulated wave fields can be generated with customized parameters tailored to specific inversion algorithms^31,32,37^. Phantom data is often used as a real-world validation tool, as the biomechanical properties estimated by inversion algorithms can be directly compared to those measured using standard mechanical testing protocols^38^. Soft tissue data can be obtained from either *ex vivo* or *in vivo* sources. *Ex vivo* tissues, often from animals, serve as a preliminary validation step before applying the algorithms to human studies. *In vivo* human MRE data primarily focus on two organs: the liver and the brain^26,29,30,34^. However, acquiring phantom and tissue MRE datasets requires access to MR scanning facilities and MRE systems, which can be a barrier for research groups focused solely on algorithm development. Additionally, when comparing different algorithms, it is more efficient to retrieve and analyze reported data rather than reconstructing an entire MRE scanning system from scratch. A representative publicly available dataset is the BioQIC server (https://bioqic-apps.charite.de/), which contains comprehensive MRE data from both phantom studies and organ examinations.

To facilitate data sharing for the development of MRE inversion algorithms and to advance the field of MRE research and biomechanical parameter inversion, we are providing a comprehensive dataset for the research community. This dataset includes wave field images, inversion results, and the corresponding inversion algorithms. The wave images were acquired using 3T scanners, ensuring high-quality data. Phantom data are included as a benchmark for validation, while *in vivo* human liver and brain data are also provided to support broader applications. This initial release provides one representative sample per category as a pilot demonstration. Subsequent versions will expand the dataset with larger sample sizes for each category to facilitate comprehensive testing and development.

## Methods

### Phantom preparation

Gelatin phantom that has mechanical properties close to human soft tissues are commonly used in elastography studies for experimental validation. Here we provide one set phantom data with inclusions^39^. The phantoms were made with agar to modulate the stiffness^38^.

**Fig. 1:**
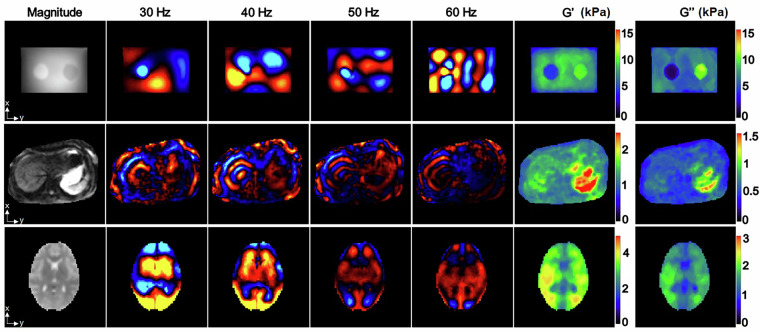
Representative anatomical, wave propagation, and modulus images for phantom, liver, and brain datasets. The first row presents agar phantom results, followed by liver (second row) and brain (third row) data from healthy volunteers. The first column shows MRE magnitude images derived from the MEG z-axis at the first phase offset (60 Hz). Columns 2-5 display first principal component displacement maps at respective vibration frequencies, while column 6 and 7 present TWENN-estimated storage modulus (G’) and loss modulus (G”).

The phantom with two cylindrical inclusions were made of agar in a plastic cubic box with dimensions of 17 cm × 12 cm × 14 cm. Both inclusions had diameters of 3.5 cm and heights of 10 cm. The concentrations of the agar for the background and the two inclusions were 1%, 0.75%, and 1.5%, respectively (Fig. 1)^40^.

### Healthy volunteer

For brain and liver MRE, the study protocols were reviewed and approved by the Institute Review Board of Shanghai Jiao Tong University (E20240268C, E20230074I). The research was conducted in accordance with the principles embodied in the Declaration of Helsinki. Informed consent was obtained from each participant, who consent to participate and have data openly shared.

### Data acquisition

All of the MRE data sets were acquired using a 3.0 T MRI system (uMR790, UIH Healthcare, Shanghai, China). Electromagnetic actuators were used for producing shear waves to the phantom, liver, and brain^39,41^. A 24-channel head coil was employed for brain and phantom imaging, with an 8-channel abdominal coil used for liver studies. An indirect actuator, driven by a function generator and power amplifier, was utilized for brain and phantom examinations^39^. The MRI console triggered the function generator to produce sinusoidal signals (0.5 V phantom; 1.5 V human) upon scan initiation, amplified by a consistent 2 × factor. Motion encoding efficiencies measured 1.30 × 10^5^ rad/mm (30 Hz), 1.70 × 10^5^ rad/mm (40 Hz), 1.36 × 10^5^ rad/mm (50 Hz), and 1.14 × 10^5^ rad/mm (60 Hz). The imaging parameters are summarized in Table 1. For liver imaging protocols, a 2 mm inter-slice gap was implemented in the acquisition. While this non-contiguous sampling strategy improves scan quality for each slice, it may compromise the accuracy of modulus quantification when employing 3D inversion algorithms.

**Table 1:** Scanning parameters for phantom, liver, and brain.

Parameters	Data types		
	Phantom	Liver	Brain
Sequence	SE-EPI	SE-EPI	SE-EPI
TE (ms)	65	54.0	65
TR (ms)	2000	1000	4000
Matrix	80 $\times$ 80	128 $\times$ 128	80 $\times$ 80
Resolution (mm^2^)	3 $\times$ 3	2.34 $\times$ 2.34	3 $\times$ 3
Slice Thickness (mm)	3	10	3
Slice Gap (mm)	0	2	0
Number of Slices	20	10	40
Frequency (Hz)	30, 40, 50, 60	30, 40, 50, 60	30, 40, 50, 60
Encoding Direction	x, y, z	x, y, z	x, y, z
Breath Control	No	Breath-hold	No

SE-EPI: spin echo-based echo planar imaging.

The phantom’s vibration plate was positioned at the container base. For brain imaging, the driver was placed laterally with its plate beneath the neck of a supine 27-year-old male volunteer, simultaneously providing neck support. Liver imaging positioned the plate on the right chest wall at xiphisternum level for a supine 25-year-old male.

### Data processing

We developed an algorithm for processing raw magnetic resonance elastography (MRE) data to generate shear modulus maps. This algorithm integrates an optimization-based phase unwrapping technique with Dual Data Consistency (Dual-DC) and a physics-informed Traveling Wave Expansion-Based Neural Network (TWENN)^34^. The Dual-DC method simultaneously addresses phase unwrapping and principal component extraction through an adaptive optimization framework that enforces dual constraints: cross-image phase consistency for motion accuracy and true phase gradient alignment for continuity preservation. This achieves unprecedented robustness against noise-induced artifacts while eliminating background phase offsets through iterative refinement. For modulus inversion, TWENN establishes a complex-valued deep learning architecture trained on physically realistic wavefields generated via Traveling Wave Expansion (TWE) model. By using displacement covariance matrices as multidimensional input descriptors, the network captures intrinsic wave propagation characteristics across varying noise levels and tissue heterogeneity. The integrated pipeline enables end-to-end transformation of wrapped phase images into quantitative viscoelastic maps.

The TWENN inversion method uniquely merges physical principles with data-driven learning, resulting in exceptional generalizability, noise resilience, and high accuracy. Key features of TWENN include: (1) User-configurable fitting window dimensions, adaptable to data quality and modulus heterogeneity; (2) Compatibility with both 2D and 3D spatial inversion modes; (3) Flexibility to process single- or multi-MRE datasets; (4) Support for diverse frequency combinations and arbitrary spatial resolutions, including anisotropic grids; (5) Customizable training protocols with controlled noise injection ratios. This versatility enables robust and reliable modulus estimation across a wide range of applications, including simulation-derived data, phantom studies, and *in vivo* human research.

## Data Records

The entire dataset is publicly accessible via the Science Data Bank^42^ (https://www.scidb.cn/en/s/eYFnie), which includes anatomical images, wave images, and inversion results. Anatomical and wave images are stored in **.mat** format, and the TWENN algorithm was employed for all inversion processes^34^. The dataset comprises three types of MRE data:

1. Agar Phantom Data: Generated from a self-made agar phantom containing two inclusions—one hard and one soft—embedded within the gel.
2. Brain Data: Acquired from the brain of a healthy volunteer.
3. Liver Data: Collected from the liver of a healthy volunteer.

All datasets were acquired using four vibration frequencies (30 Hz, 40 Hz, 50 Hz, and 60 Hz), with slice acquisition performed along the z-axis. The data are organized into four distinct categories with the following structure:

1. PhaseRaw (Raw phase data)
  - Unit: Radians
  - Dimensions: [x, y, z, phase offset, displacement direction, vibration frequency]
  - Note: For each acquisition, four phase offsets were recorded, and each phase offset includes two displacement encodings (positive and negative). Therefore, there are a total of 8 phase offsets data, among which the odd-numbered ones are positive encodings and the even-numbered ones are negative encodings.

2) U (Displacement field)

- Unit: Radians
- Dimensions: [x, y, z, phase offset, displacement direction, vibration frequency]

3) Mag (Magnitude images)
  - Dimensions: [x, y, z, phase offset, displacement direction, vibration frequency]
  - Note: For each acquisition, four phase offsets were recorded, and each phase offset includes two displacement encodings (positive and negative). Therefore, there are a total of 8 phase offsets data, among which the odd-numbered ones are positive encodings and the even-numbered ones are negative encodings.
4) G (Complex shear modulus)
  - Unit: Pascals (Pa)
  - Dimensions: [x, y, z]
  - Note: the real part represents the storage modulus and the imaginary part represents the loss modulus.

## Technical Validation

**Fig. 2:**
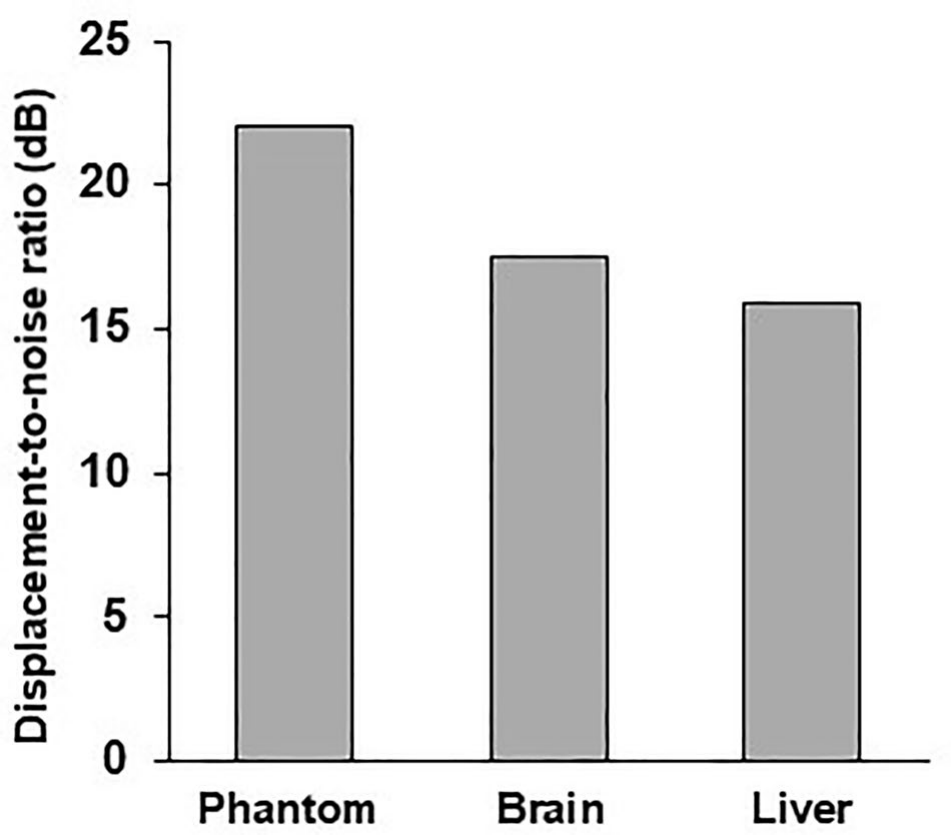
The mean displacement-to-noise ratio results of the phantom, brain, and liver dataset.

The quality of the wave images was evaluated using the displacement-to-noise ratio (DNR). The DNR quantifies the noise level in the displacement field measured by magnetic resonance elastography (MRE)^35^ and serves as a metric for comparing the effects of different MRE acquisition methods or systems^41,43^. For liver imaging, the typical mean DNR values range between 13 and 17^41^. In this dataset, displacement field data for phantom, brain, and liver are provided, with DNR results demonstrating consistent quality. Specifically, the DNR values are 22.02 dB for the phantom, 17.55 dB for the brain, and 15.86 dB for the liver, respectively (Fig. 2). In addition, the number of voxels per wave length that quantifies the spatial sampling density were also calculated^44^. The mean values and standard deviations for phantom, liver, and brain across the four frequencies were 20.75 ± 6.33, 9.09 ± 2.77, and 13.12 ± 4.00 voxels/wavelength, respectively. These results highlight the robustness and reliability of the acquired data across different sample types.

## Usage Notes

The MRE Preprocessing software is a command-line tool designed for processing magnetic resonance elastography (MRE) data. It integrates the Dual-DC phase unwrapping method and the TWENN modulus inversion framework. The tool is optimized for Linux (Ubuntu 18.04) systems with NVIDIA GPU support and offers three distinct workflow modes:

1. Phase Unwrapping Only: Converts raw wrapped phase data into complexed value wavefields.
2. Modulus Estimation Only: Generates shear modulus maps from complexed value wavefields.
3. Full Pipeline: Processes raw wrapped phase data directly into shear modulus maps.

Key configurable parameters include noise level adjustment (noise_density) and inversion kernel dimensions (inversion_kernel). The software supports both 2D and 3D inversion, accommodates variable resolutions, and incorporates adaptable noise modeling to suit diverse scenarios, such as simulations, brain studies, and liver studies.

Input and output files adhere to MATLAB format specifications for wavefield and modulus data. To facilitate implementation, a demonstration script (Pipeline_demo.m) is provided, leveraging GPU acceleration to optimize performance.

## Data Availability

The entire dataset is publicly accessible via the Science Data Bank^42^ (https://www.scidb.cn/en/s/eYFnie), which includes anatomical images, wave images, and inversion results. Anatomical and wave images are stored in **.mat** format, and the TWENN algorithm was employed for all inversion processes^34^. The dataset comprises three types of MRE data: Agar Phantom Data, Brain Data, and Liver Data.

## Code availability

The software used for the inversion is available at the Science Data Bank^45^ (https://www.scidb.cn/en/s/rEnyAb).
